# The romanticisation of mental health problems in adolescents and its implications: a narrative review

**DOI:** 10.1007/s00787-025-02701-0

**Published:** 2025-04-12

**Authors:** Awa Ndour, Lucy Foulkes

**Affiliations:** University of Oxford, Oxford, UK

**Keywords:** Romanticisation, Mental health, Adolescents, Online

## Abstract

Romanticisation is the perception and portrayal of a phenomenon as more attractive, interesting, cool, profound or desirable than it really is. There are concerns that mental health problems are increasingly romanticised, particularly among adolescents, but there is limited research on this topic. This narrative review investigated: (1) what romanticisation is in the context of adolescent mental health problems, (2) why adolescents might romanticise mental health problems, (3) the implications of romanticising mental health problems in adolescence, and (4) what interventions might reduce this phenomenon. Sixty-one publications were reviewed, including qualitative and quantitative analyses, cross-sectional and longitudinal self-report studies and conceptual reviews. Most investigated romanticisation of mental health problems online, with most researchers situated in a Western context. Identity formation, popular media influences and peer influences arose as potential explanatory factors. Negative outcomes to romanticisation were indicated, including the reinforcement of mental health problems and reduced help-seeking; few interventions to reduce the phenomenon have been proposed to date.

## Introduction

Romanticisation is the unrealistic belief, perception, or representation of something to be more desirable or attractive than it really is [55]. Objects of romanticisation are presented as idyllic, aesthetic, trendy, or interesting, while negative aspects are minimised or dismissed. In recent years, there has been growing concern that mental health problems—defined here as mental disorders and their subclinical symptoms—have been increasingly romanticised in Western countries, particularly among adolescents [34, 80, 83]. Examples can easily be found on social media platforms such as Instagram, Facebook, Twitter, TikTok and Tumblr, which show aesthetically-pleasing photos and videos of people crying, bleak landscapes, or images of severe weight loss and self-harm, accompanied by song or movie quotes, or poetic captions that express suffering and mental distress [14, 80]. The concern is that this romanticisation can spread misinformation and trivialise the difficulties experienced by people with mental health problems, ultimately increasing distress for these individuals [47, 86, 87].

In the past two decades, there have been extensive efforts led by charities, public health bodies and popular media to reduce the stigma associated with mental health problems [94], and these efforts may have inadvertently contributed to romanticisation of these difficulties. Many people now share their personal experiences of mental health problems online, including celebrities, medical professionals and lay people [37, 46, 77]. These personal accounts of mental health problems are often rewarded with praise and attention in the form of ‘likes’, comments and followers, which are powerful indicators of social acceptance [63, 85]. This form of reward might then lead social media users to draw an association between their own mental health problems and positive social outcomes [34].

Most concerns about romanticisation of mental health problems focus on adolescents [14, 80], and there are a number of factors relating to social cognitive development during this period that indicate they may be especially likely to romanticise mental health problems. Adolescents use the internet, and particularly social media, to seek emotional support and learn about mental health [78, 98], this increases the likelihood of exposure to romanticised content that may influence their attitudes and behaviour. Adolescents are more susceptible to social influence than adults, including conforming to the attitudes and behaviours shown by their peers [40, 59]. They may also be more sensitive to social reward, meaning that the social currency of likes, comments and follows are especially motivating for them [39]. This may lead them to romanticise mental health problems online to seem ‘cool’ and gain attention and support from peers [34, 80]. Lastly, adolescence is a critical period of identity formation, where a sense of belonging with peers is crucial for developing a positive sense of self [79]. Adolescents also use social media to support their identity development [45]. Mimicking the romanticisation that they see online might help adolescents belong to a social group, which in turn contributes to their developing sense of self. However, these are inferences based on theory and evidence of adolescents’ social cognitive development; to date, there have been limited attempts to review research that more directly examines romanticisation of mental health problems at this age.

In particular, it is critical to understand not just why adolescents might romanticise mental health problems but what impact it might have on them. Romanticisation may have some benefits—some individuals may feel validated or understood by hearing others talk about mental health problems in this way, and may romanticise themselves to make sense or cope with the issue [19, 50]. However, in parallel, there are concerns that romanticisation may have negative effects. For example, there is the concern that romanticisation is the problematic opposite of stigmatisation; the reversal of stigma taken to an unhelpful extreme [86, 87]. Stigmatisation and romanticisation are both misinformed perceptions, and either can prevent people with mental health problems from being understood and treated fairly and sympathetically [86, 87]. There is concern that romanticisation makes mental health problems appear trivial by dismissing their severity or their functional impact, which can make those who do not romanticise their mental health problems feel misunderstood and isolated [47]. Other evidence suggests that romanticisation could lead to negative judgement from others, especially from adults judging adolescents’ online behaviour [73, 100, 112]. Lastly, romanticisation might discourage help-seeking, both in those who romanticise their mental health problems (because they feel help is not needed), and in those who do not (because they are concerned they will not be taken seriously; [86, 87]. However, to date, a comprehensive assessment of these possible consequences and implications of romanticisation is lacking in the literature.

In this paper, we present a narrative review of research examining the romanticisation of mental health problems among adolescents. This method was chosen due to the lack of consistent terminology used to describe romanticisation, and inconsistencies in the methodologies used to explore the phenomenon. The review aimed to answer the following research questions:

1. What is romanticisation in the context of adolescent mental health problems?
2. Why might adolescents romanticise mental health problems?
3. What are the implications of the romanticisation of mental health problems in adolescence?
4. What are the interventions, if any, that can reduce romanticisation of mental health problems in adolescence?

## Method

Two searches were conducted in December 2023, on four databases: PubMed, APA PsycNet, JSTOR and Google Scholar. We first conducted a search of PubMed, APA PsycNet, JSTOR with the first three queries below in the title and abstract, combined with the Boolean operator ‘AND’. The second search included a fourth query regarding intervention, also added with ‘AND’, and searched for in the full text. This search was run to identify papers relevant to the third and fourth research questions, about the implications of romanticisation; it was run separately to avoid inappropriately narrowing the search for papers addressing the first and second research questions. Separately, an advanced Google Scholar search was conducted with all terms entered into the section labelled ‘with at least one of the words’, without Boolean operators or asterisks as the engine provides this function automatically. These terms were searched for in the publications’ titles, which were screened along with the abstracts.

The search terms were as follows:

1. (romantici*) OR (glorif*) OR (aesthetici*) OR (ideali*) OR (glamori*) OR (pro)
2. (adolescen*) OR (teenage) OR (youth)
3. (mental illness) OR (mental health) OR (self-injury) OR (depress*) OR (anxiety) OR (self-harm) OR (disorder)
4. (treatment) OR (therapy) OR (intervention)

All searches also included the terms (athlete) and (immun*), combined with the Boolean operator ‘NOT’; this was done to prevent results that discussed idealisation in terms of athlete body image and resulting mental health problems, or results discussing pro-inflammatory immune responses and depression. Both were added after initial searches returned many papers focused on these topics that were irrelevant to our research questions.

**Fig. 1:**
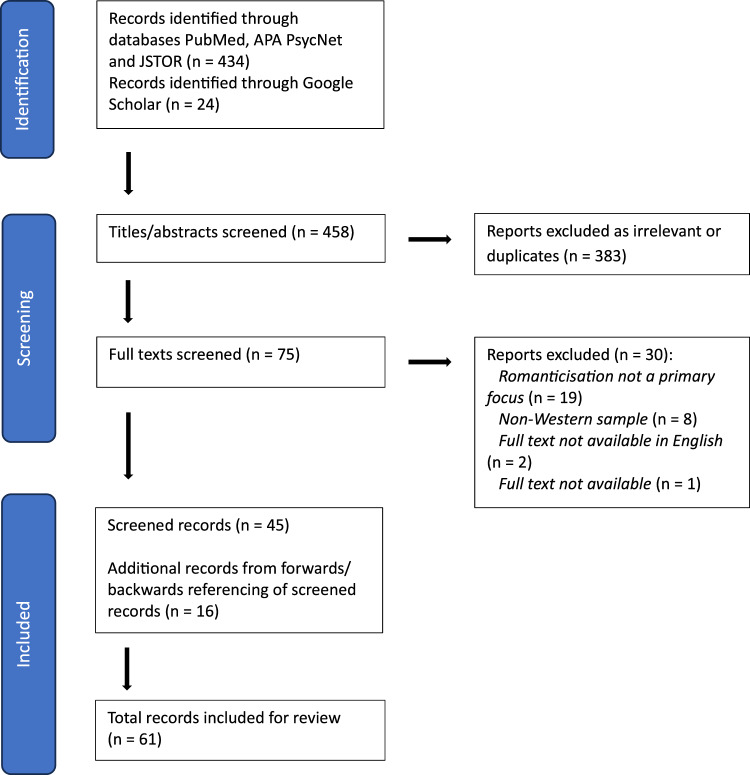
PRISMA flow diagram for literature search

After all screening was completed for papers identified in database searches (see detail below), we applied forwards and backwards referencing to the papers that met inclusion and exclusion criteria. For forwards referencing, the inclusion and exclusion criteria were applied to all forward-referenced article titles and abstracts. Publications were followed up for backwards referencing if they were cited as a relevant finding in the publications already included; their titles and abstracts were then also screened using the inclusion and exclusion criteria. See Fig. 1 for the complete search process.

Publications were included in the review if they were written in English and described or investigated any of the following: (a) representations of mental health problems that matched our understanding of romanticisation (the unrealistic belief, perception, or representation of a mental health problems to be more desirable or attractive than it really is); (b) positive attitudes towards mental health problems; (c) the encouragement of thoughts, feelings or behaviours associated with mental health problems (e.g. self-harm). Publications were excluded if the adolescent population was not discussed, either in relation to mental health problems, romanticisation, or both. Publications were also excluded if they described non-Western samples, due to potential differences in how other cultures may define adolescence [82] and in attitudes towards mental health that may affect how romanticisation manifests, if it is present [3, 53]. There were no restrictions on publication date. See Table 3 for full list of excluded publications.

## Results

A total of 61 articles were included for review (see Table 1). Publication year ranged from 2005 to 2023. Methodologies included conceptual reviews and essays, systematic reviews, content analyses (qualitative and quantitative), case reports and primary research, both cross-sectional and longitudinal. Most researchers were situated in a Western context. When the age of participants was stated, e.g. in primary research articles, the age range was 10–42 years; these studies were either conducted exclusively on participants aged 25 and under (10 articles), or included these young participants along with older participants (11 articles). Articles that did not provide a specific participant age range (40 articles; e.g. conceptual reviews, analyses of anonymous social media data), emphasised a focus on adolescents in the title and/or throughout the article. Most research used mixed sex samples, although some exclusively discussed female samples [6, 95, 96, 107] and some (such as those analysing social media content) were unable to gather demographic information. Below, we describe the findings of the review as related to each research question.

**Table 1:** Characteristics of publications included in the review

References	Research Method	Object(s) of romanticisation	Measure(s)	Participant age (years)	Main findings
Allison et al. [2]	Conceptual article	Eating disorders	N/A	N/A	Attributes pro-ED attitudes to peer influence and the idealisation of thinness. Peer groups must become a stronger focus in early clinical intervention, with strengthening of pro-recovery friendships and direct peer involvement
Arseniev-Koehler et al. [5, 6]	Quantitative content analysis	Eating disorders	N/A	15–25*	Most content from pro-ED Twitter profiles would reference a specific ED, identification with the disorder, and ED-related (‘compensatory’) behaviours. Followers of these profiles were also more likely to post pro-ED content. These communities are suggested as a focus for further investigation but warns against censorship
Baker and Lewis [8]	Thematic analysis	Self-harm	N/A	N/A	Reactions to online self-harm images (ranging in severity), mostly from people who had some connection to self-harm themselves, were positive and negative. Almost half claimed the posts were helpful, while the others claimed they were not helpful and triggering
Batterham et al. [12]	Cross-sectional primary research	Suicide	SOSS	18–30	The SOSS is established as a valid and reliable measure in a large sample of university students and staff; scores are highest on the isolation/depression scale. High stigma and glorification/normalisation scores were less common but did occur
Bates [11]	Qualitative content analysis	Eating disorders	Metaphor Identification Procedure (Pragglejaz Group 2007)	N/A	Metaphorical self-descriptions in a large sample of pro-ED profiles from the same online community fell into four categories: self as space, self as weight, perfecting the self, and the social self. These all refer to different ways in which metaphors are used by those who engage in pro-ED communities to make sense of themselves and their issues
Biddle et al. [13]	Website evaluation	Suicide	N/A	N/A	From searches of websites giving instruction or information about suicide. Many encouraged suicides, providing information on specific methods, and there was variation in the content’s relevance to the search term. The need for a balance between freedom of speech and controlled access to these sites
Blair and Abdullah [15]	Thematic analysis	Depression, anxiety, eating disorders, self-harm	N/A	N/A	Instagram posts under #depression and #anxiety followed three key themes: hashtag hijacking (using mental health hashtags for their popularity), promotion of negative behaviours, and mirroring of attitudes and behaviours
Borzekowski et al. [17]	Systematic content analysis	Eating disorders	N/A	N/A	Websites found from pro-ED search terms were mostly interactive: characteristics included sharing tips and motivation, some recovery content, rejection of performative ED, and religious metaphors. The dangers of these social interactions are discussed
Calear et al. [21]	Cross-sectional primary research	Suicide	Stigma of Suicide Scale-Short Form (SOSS; [12]) Literacy of Suicide Scale-Short Form (LOSS; [21])	11–17	Rates of glorification/normalisation were generally low among Australian youth. High scores were related to low suicide literacy levels, high levels of suicidal ideation, and exposure to suicidal thinking in friends. This is suggested to negatively impact help-seeking
Carmichael and Whitley [22]	Qualitative thematic analysis	Suicide	N/A	N/A	Canadian news reports on’13 Reasons Why’ generally adhere to *Mindset*’s suicide reporting guidelines, and critically discuss the show’s romanticisation of teen suicide and the implications for young people. Further steps could be taken to signpost educational and supportive resources
Cavazos-Rehg et al. [23]	Qualitative thematic analysis	Depression, suicide, self-harm	N/A	14–20*	Posts on Tumblr discussing mental illness had themes of self-hatred, loneliness, and suicide/death. Graphic images were common. Both positive and negative implications are discussed, with the recommendation that parental and professional oversight is required in cases where mental illness is encouraged and glorified
Chan and Sireling [24]	Conceptual article and case report	Bipolar disorder	N/A	N/A	Discusses the rise in cases of people wanting bipolar diagnoses, and self-diagnosing, following the stories of several celebrities talking about their diagnoses in a positive manner. Psychiatrists are suggested to be sensitive to patient opinions and emphasise the negative consequences of receiving a diagnosis to them
Chancellor et al. [26]	Quantitative content analysis	Eating disorders, self-harm	Latent Dirichlect Allocation (LDA; Blei et al. 2003) for Mental Illness Severity	N/A	A new LDA model for estimating and predicting future mental illness severity is validated. Pro-ED content posters on Instagram had increasing mental illness severity over time. Platforms must increase social support, warning systems, and use the LDA model to identify high-risk cases
Chancellor et al. [27]	Longitudinal quantitative content analysis	Eating disorders	N/A	N/A	Pro-ED posts on Instagram that end up getting removed are associated with higher risk of self-harm and suicide. Posts likely to be removed can often be identified through the tags used—the use of the same classifier is recommended for social media platforms and intervention leaders
Chancellor et al. [28]	Longitudinal quantitative content analysis	Eating disorders	N/A	N/A	Pro-ED posts on Instagram use ‘lexical variants’ (non-word variations that keep the same meaning) in tags to avoid being removed. This is attributed to maintaining a sense of community and feeling targeted for self-expression, particularly for young people. Platforms are advised to increase the exposure to recovery content and attempt to pre-emptively detect these lexical variants
Chancellor et al. [25]	Quantitative content analysis and a validation study	Eating disorders, self-harm	Deep Convolutional Neural Network (CNN; Krizhevsky 2012) for image classificationSkip-gram and word2vec models for text embeddings	N/A	A new classification system using image and text analysis is validated for identifying pro-ED and pro-self-harm content on Tumblr that violates community guidelines. While the approach is successful, it is acknowledged that consistent monitoring of the systems criteria by professionals, parents, and stakeholders is necessary for a sensitive approach that does not remove freedom of speech and self-expression
Custers [30]	Conceptual article	Eating disorders	N/A	N/A	The presence of pro-ED content online is increasing, and control over it is decreasing—this is particularly true for content created by and for adolescents. Evidence for the negative outcomes and reinforcement of symptoms is outlined. Clinicians are advised to directly confront and monitor patients’ online activities
D'Agati et al. [31]	Monograph	Suicide	N/A	N/A	The YA novel’13 Reasons Why’, and its Netflix-adapted TV series misrepresent suicide as a consequence of adverse life events with no mention mental illness. It also glamorises suicide by giving the victim power and romantic appeal post-suicide. The series, intended for a teenage audience, is suggested to increase adolescent reluctance to seek help as it implies that parents, teachers, and mental health professionals are incompetent
Day and Keys [32]	Website evaluation	Eating disorders	N/A	N/A	Pro-ED websites included personalised portrayals of the disorder that subvert conventional (clinical) understandings of the illness—EDs are instead presented as a lifestyle choice, part of individual identity, and as a means of gaining control. Findings are discussed from a feminist perspective
De Choudhury [33]	Qualitative and quantitative content analysis	Eating disorders	N/A	N/A	Pro-ED Tumblr posts often presented ED-related behaviours as lifestyle choices, had aggressive tones, appeared overly extroverted and focused on the self, and included more images than pro-recovery posts. Suggestions for intervention involve confronting attitudes towards the disorder and body image, and more thorough and constructive content warnings
Del Carpio et al. (2020)	Longitudinal primary research	Suicide	CASE study questionnaire (Hawton 2006) Self-Injurious Thoughts and Behaviours Interview (STBI; Nock 2007) Defeat and Entrapment scales (Gilbert and Allan 1998) Brief COPE (Carver 1997) Rosenberg Self-Esteem Scale (Rosenberg 1965) Multidimensional Scale of Perceived Social Support (MSPSS; Zimet 1988) SOSS [12]	11–17	High glorification/normalisation scores were associated with self-harm group membership both concurrently and longitudinally; the same was found for suicidal ideation, and bereavement by suicide. Suggestions are made to direct more efforts to address beliefs about suicide
Dunn [34, 35]	Thesis	Mental illnesses (general)	N/A	N/A	Highlights films and TV as a primary means of both de-stigmatisation by humanising people with mental illnesses, which can lead to romanticisation. Social media and pop culture media targeted towards teenagers are also romanticising mental illness more and more
Durkee et al. (2011)	Narrative review	Suicide	N/A	N/A	Literature discussing how suicide is addressed on the internet have some common themes: pathological internet use associated with suicidality, exposure to pro-suicide websites, suicide pacts made on the Internet (‘net suicides’), and Internet suicide prevention. Pro-suicide websites present suicide as a problem-solving strategy and a personal right. Exposure to pro-suicide websites, and net suicide behaviours, were both associated with higher risk
Dyson et al. [36]	Systematic review	Self-harm	N/A	19–21*	There was mostly qualitative research on discussion forums and some social media platforms (Myspace, Facebook, YouTube). Support was provided online, like suggestions for treatment and how to stop self-harming. Risks included normalising and accepting self-harm, triggering content, and sharing suicidal plans
Fitzsimmons-Craft et al. [38]	Cross-sectional primary research	Eating disorders	Unspecified questionnaire Stanford-Washington Eating Disorder screen (Graham 2019) Eating Disorders Quality of Life instrument (Engel 2006) PHQ-9 (Kroenke 2001) GAD-7 (Spitzer 2006)	15–25	Exposure to body image content (‘thinspiration’) was most common on Instagram among both adolescents and young adults. There were generally high levels of exposure across social media platforms and age, and this was associated with a strong prevalence of clinical or subclinical ED in participants. Engagement with pro-ED content is discussed as a symptom of EDs. A significant lack of treatment or problem recognition is also observed
Fox [41]	Virtual participant observation	Eating disorders	N/A	14–42	Participants on a pro-ED website/forum expressed views that subvert medical models of anorexia, but also paradoxically subvert social models too. Regardless, they view anorexia as a coping mechanism for psychological/social stressors which does not need to be cured (anti-recovery)
Franzén and Gottzén [42]	Website evaluation	Self-harm	N/A	15–28*	Normalising (connecting self-harm to beauty and resilience) and pathologizing (connecting to repulsion and moral inferiority) discourses were found in a Swedish online self-harm community. A co-occurrence of both viewpoints is associated with ‘authenticity’ as a self-harmer
Fung Chun-Hai et al. (2020)	Systematic review	Eating disorders, depression	N/A	N/A	Health-related messages on image-based social media platforms (Instagram, Pinterest, Tumblr, Flickr) differ depending on the health issue being discussed. For mental illnesses, the ‘echo chamber’ effect (where communication mostly occurs between like-minded people who reinforce each other’s beliefs) is found in pro-ED Flickr communities
Gould et al. [43]	Quantitative and qualitative content analysis	Suicide	Qualitative Content Analytic Abstract Form [43]	N/A	There was generally a high degree of agreement between raters was found in the use of a new content analytic approach for news coverage of suicide. Suicide was sensationalised more than it was glorified or romanticised, but all three were observed and identified as risks for suicide contagion
Hajdu [48, 48]	Systematic review, primary research, and thematic analysis	Suicide, self-harm, depression, eating disorders	N/A	18–21	(1) Sharing content on suicide and self-harm is related to higher risk than the viewing of such content. (2) The theme of mental illness being a desirable social norm arose from semi-structured interviews. Suggested countermeasures include co-written, more personal advisory messages for social media platforms and websites, and social media monitoring for professionals
Harness and Getzen [49]	Conceptual article	Mental illness (general)	N/A	N/A	There has been a rise in young people adopting mentally ill identities on TikTok, particularly post-COVID. Guidelines for clinicians and caregivers involve improving sympathetic education on mental illnesses, understanding the causes of this phenomenon, and exploring adolescent social media use
Hilton [51]	Thematic analysis	Self-harm, eating disorders	N/A	*M* = 17.54*	Themes identified from Twitter posts were celebrity influence (both positive and negative), negative attitudes towards self-harm jokes, social support, eating disorders and self-harm, videos and personal stories
Inayat et al. [54]	Thematic analysis	Mental illnesses (general)	N/A	N/A	Six YA novels have been criticised as romanticising mental illness by making mental illness an attractive trait that plays a role in their romantic relationships. They appear to use mental illness as nothing more than a plot point and facilitate the belief that professionals and adults are incompetent in providing support. Negative effects on help-seeking behaviours are discussed as an outcome
Jacob et al. [57]	Primary research and thematic analysis	Self-harm	N/A	16–24	Many participants report previously engaging with online pro-self-harm content because of pre-existing behaviours, which reinforced their self-harm due to normalisation and sharing of techniques. Participants appear to be self-conscious of any glorifying behaviours, and connections to subcultures like goth and emo are discussed
Keipi et al. [58]	Primary research	Suicide, self-harm, eating disorders	Unspecified questionnaires (× 3) Subjective Well-Being scale (Veenhoven 2012)	15–30	A considerable number of participants had been exposed to online content that promoted suicide, self-harm or ED-related behaviours. This was also associated with lower subjective wellbeing. Cultural differences between the USA and Finland are discussed, giving implications for the effects of globalisation
Lewis and Arbuthnott (2012)	Quantitative content analysis	Eating disorders	N/A	N/A	The most common search terms for eating disorder content on Google were ‘pro-ana’ and variations of ‘thinspiration’, which were associated with content with higher harm scores and encouragement of eating disorders. Intervention and re-direction in the search results stage is suggested
Lewis and Seko [62]	Systematic review and thematic analysis	Self-harm	N/A	N/A	Four benefits and three risks were identified in the literature for online self-harm-related activities. Benefits included mitigating social isolation, encouraged recovery, disclosure of emotions, and suppressing self-harm urges. Risks included self-harm triggering, self-harm reinforcement, and stigmatisation
Logrieco et al. (2021)	Case study report	Eating disorders	N/A	14	This adolescent was hospitalised due extreme weight loss and was diagnosed with anorexia nervosa and reported engaging in these behaviours because of a desire to have an extreme experience like her peers, instead of a desire to lose weight like many other eating disorders (EDs). She reports being inspired by pro-ED content on TikTok, despite there being considerable ‘anti pro-ED’ posts and censoring of pro-ED posts
Lyons et al. [64]	Quantitative content analysis	Eating disorders	N/A	17–21*	The language used on pro-anorexia websites had ‘hedonic’ focus on positive emotions, less cognitive focus, and less occupation or focus on the self, compared to pro-recovery posts. This is suggested to be an emotional coping strategy, and linguistic signs like these can be used for early identification of eating disorders
Mento et al. [66]	Systematic review	Eating disorders	N/A	N/A	Most research on pro-ED internet content focused on female adolescents and found them to gain a feeling of support from these sites. ‘Problematic internet use’ of this nature was predicts reinforced ED symptoms, but this may be caused by sensation-seeking and a need for social support in adolescents with EDs
Minkkinen et al. [67]	Cross-sectional primary research	Suicide, self-harm	Unspecified questionnaire^a^	15–30	Victimization (online and offline) were related to high exposure to pro-self-harm and pro-suicide websites, often together. Promoting closer connection to primary groups (that balance emotional distress) is suggested as a countermeasure
Moyer et al. [68]	Website evaluation	Self-harm	N/A	N/A	Most non-interactive websites about self-harm for young people were informative and supportive, but some actively encouraged self-harm and had triggering content. Suggestions for evaluating websites using Health On Net guidelines are given for school counsellors
Mulveen and Hepworth [69]	Interpretive Phenomenological analysis	Eating disorders	N/A	16–35*	Selected posts on pro-anorexia websites or forums had the themes: tips and tricks, ‘ana’ vs anorexia nervosa, social support, and a need for anorexia
Norris et al. [74]	Website evaluation and thematic analysis	Eating disorders	N/A	N/A	Of the pro-ED websites identified, many used images of celebrities, quotes, and technique-sharing to actively encourage ED behaviours. The sites generally had themes of ED outcomes measuring success and beauty, social support, and ED being a lifestyle choice
Oksanen et al. (2015)	Quantitative ‘sentiment’ content analysis	Eating disorders	N/A	N/A	Pro-anorexia and anti-pro-anorexia comments on YouTube videos often contain emotional sentiments. Anti-pro-anorexia comments had more positive sentiments and had more views and likes compared to pro-anorexia comments. There was little interaction between pro- and anti-pro-ED commenters
Olan and Richmond (2023)	Monograph	Depression, anxiety, mental illnesses (general)	N/A	N/A	Both YA novels explored here are found to depict mental illness pathology fairly accurately by DSM-V standards. However, mental illness is also romanticised in the surrounding characters’ misunderstanding of the illness (creating a misunderstood hero narrative) and using mental illness as a plot point. It is argued that these portrayals may perpetuate stigmatising beliefs
Pater and Mynatta [7, 75]	Conceptual article	Self-harm, eating disorders	N/A	N/A	Researchers define digital self-harm to be online behaviours (posts, comments, and other activity) that “supports” and reinforces self-harm. Although few causal links can be drawn between digital self-harm and young people’s offline struggles, it is agreed that this content online is harmful and should be monitored
Schipper [83]	Qualitative content analysis	Mental illnesses (general)	N/A	15–29	Many young people’s TikTok’s discussing mental illness are anecdotal or humorous; they sometimes present living with mental illness as a “hero’s struggle” and utilise TikTok’s blending of media types and pop culture references to romanticise
Seko and Lewis [84]	Visual narrative analysis	Self-harm	N/A	N/A	41% of identified Tumblr posts showed direct self-harm, and 58% showed indirect self-harm. Many had pro-recovery, hopeless, denotative, or normalising narratives. Direct self-harm was reblogged less and indirect
Shrestha [86, 87]	Conceptual article	Suicide, self-harm, depression, anxiety, eating disorders, bipolar	N/A	N/A	Outlines how mental illnesses are made to be an accessory and source of pity from others on Tumblr. ‘Echo chambers’ are created in communities that share content on mental illness and reinforces them through a negative feedback loop, particularly for adolescents. Self-diagnosis is also highlighted as an outcome of romanticisation
Singaravelu et al. [89]	Website evaluation	Self-harm, suicide, general mental state	N/A	N/A	Over half the websites found with 6 search terms has supportive content that encouraged help-seeking. A concerning proportion also had advice on how to self-harm and actively encouraged it. Professionals, schools, and guardians are advised to enquire about adolescent use of these websites
Sowles et al. [90]	Qualitative content analysis	Eating disorders	N/A	17–29	Review of a large subreddit found 4 core themes in line with ED psychopathology. The content also covered feelings of success or failure surrounding ED behaviour, with responses encouraging or reinforcing these perceptions despite being quite self-aware. It is suggested that clinicians and parents are educated on these communities
Sukunesan et al. (2021)	Quantitative content analysis	Eating disorders	N/A	N/A	#thinspo and #proana are identified as the most common pro-ED tags used on Twitter, which are found to be specific to EDs and not health or fitness. A sense of community motivates posting, with following appearing to be more significant for exposure than post engagement
Tanner [95]	Photo essay	Suicide, self-harm, depression, anxiety, eating disorders	N/A	N/A	Argues that most graphic depictions of mental illnesses or symptoms can actively encourage or reinforce them when they are posted publicly. Instagram posts by adolescent girls often use photos, words, or both, to promote eating disorders and self-harm
Thelandersson [96]	Photo essay	Mental illnesses (general)	N/A	N/A	The ‘sad girl’ persona appears on Tumblr with aesthetic and humorous posts about mental illness, and constructs a shared, romanticised aesthetic. Suggestions are made to shift public perceptions of mental illness away from full psychiatric models, to incorporate mental illness as part of human experience
Turja et al. (2017)	Cross-sectional primary research	Eating disorders	Unspecified Subjective Wellbeing Questionnaire Unspecified Victimization Questionnaire	15–30	Across three of the four countries studied (US, Germany, Finland), lower subjective wellbeing was associated with exposure to pro-ED content—this relationship was moderated by self-reported social belonging in offline groups. Essentially, poorer subjective wellbeing is associated with more pro-ED exposure, but less so with a strong sense of belonging in primary groups
Wang et al. [103]	Quantitative content analysis	Suicide	N/A	N/A	News coverage on ‘13 Reasons Why’ mostly criticised the show; reporters must better adhere to WHO suicide prevention guidelines (WHO, 2017). Media producers and researchers must consider educational content that accompanies the original work
Wester et al. [105]	Conceptual article	Self-harm	N/A	N/A	A multi-tiered system of support is discussed as an effective school-based intervention to combat the social contagion of non-suicidal self-injury, to promote healthy emotion regulation, and to identify high-risk individuals
Whitehead [107]	Case report and website evaluation	Eating disorders	Virtual ethnographic approach (Hine 2000) [107]	N/A	Explores how one pro-ED community appear to have a collective identity and finds themes in their online content of promoting secrecy, accommodating patriarchal expectations, linking beauty to self-worth, and social support via ‘fandom’ methods. The site provides an informal warning that openly labels itself as a pro-ED site for people uninterested in recovery
Whitlock et al. [109]	Qualitative content analysis	Self-harm	N/A	12–47*	Despite finding many message boards that create a supportive community, there were also many message boards where self-harm techniques was actively encouraged, with most posters being 12–20 years old. Reinforcement of self-injurious behaviour and reluctance to find alternate coping strategies are discussed as potential outcomes
Wilson et al. [110]	Cross-sectional primary research	Eating disorders	Unspecified questionnaires for parents and children	10–22	Adolescents with EDs visit both pro-ED and pro-recovery sites a large amount, and parents are generally unaware of their children’s online activities, nor do they use the Internet much themselves. Pro-ED and pro-recovery websites are also found to have similar outcomes, suggesting online ED content can be harmful regardless of the intention
Yom-Tov et al. [111]	Online randomised control trial (RCT)	Eating disorders	N/A	N/A	The RCT tested adverts directing people to one of three sites: National Eating Disorders Association, National Institutes of Mental Health, and MyProAna. These appeared following searches for ‘thinspo’ or ‘pro-ana’ on popular search engines, and exposure to the adverts was associated with fewer of these searches being made. The largest reduction was with adverts for MyProAna, which is still a pro-ED site but considered as much less problematic than others

Note that ‘ED’ is an abbreviation for eating disorders, and specifically refers to anorexia nervosa and bulimia nervosa—no other types of eating disorder arose in this literature). Age ranges marked with an asterisk (*) indicate where the age of a sample has been estimated by the authors based on demographic information available online

^a^An unnamed questionnaire developed for the purpose of the specific research questions, and unlikely to be used in other research

### Research question 1: What is romanticisation in the context of adolescent mental health problems?

A variety of terminology was used in these articles to refer to the broader concept of romanticisation. For example, authors described the *glamorising*, *sensationalising* and *aestheticising* of mental health problems (see Table 2 for full list of terminology and definitions). Many papers referred to the promotion or encouragement of behaviours associated with mental health problems, particularly referencing pro-eating disorder, pro-self-harm, and pro-suicide communities [38, 58, 67]. We considered this a form of romanticisation, as it is involves encouraging others to view mental health problems in a more positive way [112]. The specific term varies depending on the way in which the mental health problem is seen as desirable (for example, because it is aesthetic, glamorous, useful, attention-grabbing, or admirable), but the terms convey the same broad process of romanticisation, i.e. the presentation of mental health problems as more positive that they really are.

**Table 2:** Terminology used to describe romanticisation

Term	Definition
Aestheticise	To make aesthetic; to make attractive or acceptable to refined taste; to make a subject of artistic treatment
Glamorise	To make glamorous or attractive
Glorify	To describe or represent as glorious; to extol, honour, magnify with praise
Humanise	To make more humane; to civilise, refine; to make more gentle or tender, soften
Idealise	To make ideal; to represent in an ideal form or character; to regard as an ideal of perfection or excellence
Normalise	To start to consider something as normal, or to make something start to be considered as normal
Romanticise	To make romantic or idealised in character; to make something seem better or more appealing than it really is; to describe, portray, or view in a romantic manner
Sensationalise	To present or report something in a sensational, lurid, or melodramatic manner

All definitions taken from the Oxford English Dictionary (2024). British English spellings used

#### How are different mental health problems romanticised

##### Suicide

Much of the research investigating the romanticisation of suicide described the glorification of suicides or suicide-related content in news coverage [43, 103, 108]. This involved sensationalised language choices in headlines (such as “shocking” or “graphic”), in descriptions of suicide attempts using “success” and “failure” phrasing, and in phrases such as “freitod” in German newspapers specifically, a term that translates to “free death” and implies suicide is a means of liberation and empowerment [4, 43, 103]. News reports also publicise celebrity suicides more than other suicides and may implicitly promote admiration of the person and their suicide by referencing their superior celebrity status [92].

Fictional entertainment was also found to romanticise suicide. Six publications discussed the young adult (YA) novel and adapted Netflix series ‘*13 Reasons Why*’, a story about the aftermath of a teenage girl’s suicide [22, 31, 54, 83, 86, 87, 103]. Commentary criticises both the novel and TV series for seeming to glamorise several positive outcomes to suicide. In the story, the victim is martyrised and empowered in her death, as her ‘suicide notes’ (taped voice messages) gives her a voice that she did not have before; the story also involves several positive changes surrounding anti-bullying and mental health awareness in her school being made in her name [31]. The story is also told through a romantic lens, as the main character (a friend and romantic interest) feels a stronger connection to the deceased girl because of her suicide notes [31].

The glorification of suicide was also discussed in the development paper of the Stigma of Suicide Scale (SOSS; [12]), which to our knowledge is the only validated measure of any form of romanticised attitudes towards mental health problems. This self-report measure asks respondents to rate the extent to which they agree with a list of adjectives used to describe someone who dies by suicide, and there are three subscales: Stigma, Isolation/Depression, and Glorification/Normalisation. The final subscale best reflects romanticisation, with adjectives such as “brave”, “powerful, and “fearless”; the scale’s validation indicates that suicide is sometimes perceived by individuals and presented by the media as admirable, sensational and as a means for achieving positive outcomes.

##### Self-harm

The romanticisation of self-harm was typically investigated via content analyses and conceptual reviews. Methods often used general search terms like ‘self-harm’ or ‘self-injury’; however, most research findings focus on self-cutting, possibly because this is the most common form of adolescent self-harm [51, 109]. Research in this area found that self-harm is romanticised because it can be actively encouraged, with adolescents sharing techniques [57, 75, 89, 109], promoting the concealment of self-harm from others [36, 62, 109], and defending it as a valid and liberating means of emotion regulation [95, 105].

Much of this research analysed pro-self-harm content online, either on websites and internet forums [8, 42, 109] or social media platforms, particularly Tumblr and Instagram [23, 48, 84]. The analysed content generally involved graphic photos of the individual’s own cuts [23, 109], although sometimes this self-harm was presented in a more aesthetically-pleasing way with poetic text, music and greyscale photos, which reduced the explicit shock factor of the photo [75, 95]. Self-harm was also found to be a social norm in some adolescent groups and was promoted online as a ‘friendship ritual’ [105, 109]. Promotion of self-harm in adolescents can therefore appear as a social behaviour, even becoming competitive at times with adolescents attempting to have the ‘most severe’ self-harm [8, 57]. Overall, self-harm is often romanticised online—adolescents use imagery and online communication to present self-harm as a visually-pleasing, beneficial, valid, and socially acceptable and rewarding behaviour.

##### Eating disorders

Conceptual reviews and content analyses also examined the romanticisation of eating disorders, often in ‘pro-ED’ content and communities online. As with self-harm content, pro-ED posts were found to involve graphic photos of extreme weight loss, paired with motivating or poetic captions that present the disorder as a necessity for achieving positive outcomes, or as beautiful and profound [30, 41, 95]. Indeed, online pro-ED content (also referred to as ‘pro-ana’ and ‘pro-mia’, for anorexia and bulimia respectively) often promotes eating disorders as an ‘alternative lifestyle choice’ [6, 27, 32, 33]. These publications described how those with pro-ED attitudes reject outside help and medical definitions of eating disorders, as they do not see it as a disorder to be treated but rather as a way of life [2, 69, 104]. Individuals in these online communities equate beauty with low body weight and thinness, achieved through self-starvation, which becomes a measure of self-worth [74, 95, 107]. In other words, research into pro-ED online communities show that eating disorders are romanticised when they are seen by adolescents, and particularly girls and women, to be a method of increasing beauty and self-worth, and a method of reclaiming control over one’s body; they may therefore be viewed as a positive means of achieving desired goals.

Similar to self-harm, there is a social element to the romanticisation of eating disorders: studies found that individuals encourage each other’s disordered eating by sharing techniques for restrictive dieting or purging [2, 33, 69, 110]. Advice and encouragement for the concealment of eating disorders were also provided in pro-ED spaces [17], and some posts were actively anti-recovery [41]. Some pro-ED content used religious metaphors to encourage eating disorders by labelling dieting and purging techniques as ‘creeds’ or ‘commandments’ [17, 30, 32, 69]. Individuals received peer support in pro-ED communities [26, 66], and would sometimes compete with their peers on weight loss [2, 66, 69]. Online pro-ED content also personifies the disorder (referred to as ‘ana’ and ‘mia’), either to present eating disorders as ‘friends’ [32], or as villains in scenario-based TikTok posts intending to be humorous or relatable [83]. In sum, the romanticisation of both self-harm and eating disorders appear in the literature to be social behaviours, with individuals offering practical encouragement of specific maladaptive behaviours, with some aesthetic elements.

##### Other mental health issues

Depression, bipolar disorder and anxiety occasionally appear as objects of romanticisation in the literature. Some papers investigated romanticised depression on Tumblr and Instagram where greyscale, aesthetically-pleasing posts depicting sadness or depression were accompanied by a poetic message or quote [48, 86, 87]. Some users would tag their post with the terms ‘depression’ and ‘anxiety’ to increase the visibility of the post [15]. These presentations portrayed the issues as attention-grabbing, interesting, and aesthetically pleasing, implying that depression and anxiety have some desirable elements. Some posts make use of pop culture hashtags, images, quotes and music to present depression as visually appealing or humorous [51, 83, 96]. Romanticised bipolar disorder appeared in a case report of two individuals who had self-diagnosed following celebrity accounts of positive experiences with the disorder [24]. The authors wrote that these celebrities discuss feelings of heightened energy, creativity, and power, and describe the disorder as a valuable part of their identity, which may have influenced the desirability of this label in some patients [24, 86, 87]. Together, this research highlights that many mental health problems are sometimes romanticised, typically online: they are associated with positive outcomes like social reward (e.g. attention, praise), stress relief (specifically for suicide and self-harm) or reaching ideal body types, and these messages are communicated among social groups.

### Research question 2: Why might adolescents romanticise mental health problems?

#### Susceptibility to social influence

In the reviewed literature, there were a number of explanations proposed for why adolescence might be a time of particular susceptibility for the romanticisation of mental health problems. The first is that adolescents are more influenced by their peers than other age groups [23, 40, 105]. Social contagion of eating disorders and self-harm among adolescents has been observed in hospitals, detention facilities, and schools [2, 65, 81, 104, 105, 109]. Some papers referenced social learning and social cognitive theory [9, 10] to explain this phenomenon, describing how behaviours like self-harm are learned in adolescence through admired peers and the perception that the behaviour is normative, leading to social contagion of the behaviour [30, 101, 105]. The reviewed literature suggests that social contagion is enhanced when adolescents are exposed to explicit positive attitudes towards the behaviour, which can also occur online [2, 61].

Other researchers highlighted the role of social belonging and its importance in adolescence. Analyses of pro-ED and pro-self-harm content online found that adolescents express a sense of acceptance and belonging when participating in these communities [2, 7, 20]. Some research indicates that adolescents might search online for what they lack offline, as a sense of belonging in primary (offline) groups was associated with less exposure to pro-self-harm and pro-suicide websites [67]. Relatedly, other research indicates that adolescents seek out this content because it offers peer validation and understanding [36, 89], group solidarity [84], and establishment of a collective identity with shared opinions [2, 32, 96], all of which are important and highly motivating aspects of adolescent social development [99].

This peer validation may lead adolescents to view mental health problems in a more positive light, i.e. to romanticise them. One author wrote that the exchange of validation between adolescents on social media occurs in ‘echo chambers’ [86, 87], where negative emotion is reinforced through acceptance, causing adolescents to cling to these communities and, by proxy, to the mental health problem that the community is based around [14]. Some adolescents are actively encouraged to continue with eating disorder behaviours [17] and others are praised for their ‘success’ in extreme weight-loss [2, 69]. Some may seek to gain pity and sympathy from peers online [86, 87], or popularity through humour and aesthetic [15, 34]. Therefore, many adolescents evidently gain some form of peer validation for discussing their mental health problems, in the form of sympathy, praise and attention; these outcomes may explain why adolescents romanticise mental health problems.

#### Pop culture influences

Another reason why adolescents might romanticise mental health problems is that they see these problems being discussed in relation to celebrities, whom they view in a positive light. In many reviewed articles, celebrities are noted as a prominent influence on adolescent attitudes and behaviours towards mental health. Reports on celebrity suicides, particularly those that sensationalise the deaths, have been associated with adolescent suicide contagion [71, 72, 91]. Pro-self-harm and pro-ED communities often idolise celebrities who have disclosed personal experiences with the same issue [60], and individuals in these communities post images of celebrities as motivation, which can induce ‘copycat’ (contagion) behaviours [17]. Analysis of Twitter posts found that celebrity fanbases sometimes defend and encourage self-harm in celebrities’ names; for example the hashtag ‘Cut4Bieber’ was used among Justin Bieber fans who wanted to post pictures of their self-harm protest against his use of marijuana [51]. Celebrities are already idealised by many young people and in the mass media [96], so associations with mental health problems may cause the mental health problems to be idealised (and therefore romanticised) by proxy.

Relatedly, adolescents are often exposed to positive representations of mental health problems via fictional entertainment. Reviews of YA novels, films and television programmes that have adolescent target audiences identified romanticised representations of mental health problems. Representations like these are often unrealistic, as they do not reflect many people’s lived experiences of mental health problems, which can involve significantly more misunderstanding and stigma and significantly less support and attention [83, 86, 87]. Young people, as the target audience for this entertainment, are more at risk of adopting romanticised beliefs from these representations. Norris and colleagues also note that relative to adults, adolescents may be less able to critically evaluate the content they are exposed to, perhaps due to their relative lack of life experience and less mature cognitive development, which may cause them to view these representations as realistic [74].

#### Identity formation

Finally, the literature indicates that adolescents may romanticise mental health problems because this contributes to their identity formation in a helpful or desirable way. Content analyses of romanticisation online find it to be a form of identity exploration and expression [6, 11, 70]. Social media posting is generally considered a rapidly changing, multi-modal form of self-expression [56, 83, 110]. Multiple content analyses found that adolescents in pro-ED and pro-self-harm communities feel the anonymity and sense of community allows them to explore their sense of self through their disorder, whereas offline spaces and social groups do not [6, 11, 15, 48, 109]. Observations of adolescents’ posts on TikTok also find them to adopt mentally ill personas or ‘roles’ [49]. As discussed, identification with one’s mental health problem was common in content analyses of online mental health content, particularly self-labelling [2, 75, 86, 87, 96]. Self-reports from adolescents engaging in pro-ED communities described the disorder as ‘all-consuming’ [41], implying that they had no choice but to identify with the disorder. Others appear to identify with a mental health problem because it offers a more helpful way of understanding themselves and their difficulties, relative to any alternatives, particularly for bipolar disorder or eating disorders [24, 86, 87]. Together, the research to date suggests that adolescents may romanticise mental health problems because, through group identification and expressions of their distress online, their mental health problems may have become an integral, helpful way of understanding their identity and accessing social support.

### Research question 3: What are the implications of romanticisation?

#### Effect on mental health

There was a consensus across much of the reviewed literature that romanticising mental health problems, regardless of what form this takes, might reinforce the individual’s mental health problem, particularly if it is behaviour-based like self-harm and eating disorders [6, 48, 51, 86, 87, 109]. A longitudinal content analysis of pro-ED Instagram profiles found that the severity of the individual’s mental health problem, indexed based on the post’s content, increased over time alongside the extent to which the posts promoted (i.e. romanticised) eating disorders, although the direction of the relationship is unclear; these two variables may have increased in parallel without necessarily having a causal relationship [27]. However, qualitative data indicates that the sharing and learning of self-harm and eating disorder techniques in online communities can make the behaviours more severe [109, 110], and the television series *13 Reasons Why* was associated with increased teenage suicide rates [18, 29], and increased use of adolescent crisis helplines [97], suggesting that romanticisation may indeed reinforce or encourage mental health problems.

#### Effect on help-seeking

Many papers reviewed here suggested that romanticisation may lead adolescents to have more negative attitudes towards help-seeking [21, 89]. Firstly, they may experience ‘us and them’ mentalities where they feel rejected and misunderstood by all except their online peer groups, thus making the individual reluctant to seek help from family members or mental health professionals in the offline world [2, 34]. Qualitative research with adolescents indicates that this mentality is reinforced by the increase in judgement, mockery and criticism from outsiders because of online romanticisation [57]. Adolescents may adopt beliefs from their peers or the media about the incompetence of professionals and adults in providing support—*13 Reasons Why,* for example, presents the victim’s parents, the school and mental health professionals as unable to understand and support the victim [31, 103]. Indeed, some may not wish to seek help at all if they believe they do not have a mental disorder, and many pro-ED communities define themselves as being uninterested in recovery [90, 107]. Many adolescents wish to continue romanticising their mental health problems instead: analysis of some pro-ED Instagram profiles found frequent use of ‘lexical variants’ (hashtags with non-words that preserve the original meaning, such as “thyghapp” instead of “thigh gap”) to bypass content moderation schemes [28]. These profiles had more engagement from other users with lexical variants than with regular hashtags, and these variants increased with complexity over time, possibly to ensure continued engagement [28].

Some research noted the potential beneficial effects of romanticisation, particularly that it might be associated with more social support for adolescents with mental health problems. Specifically, research has found that the sense of community felt by individuals in online spaces that romanticise, particularly in pro-ED forums, can make the individual feel better supported than in professional therapeutic settings [19, 111]. This was suggested to result from an increased level of understanding and acceptance coming from peers in these romanticising communities. Similarly, in an interview study of young people who have self-harmed, participants claimed that viewing romanticised posts online, particularly on Tumblr, made them feel less alone, more understanding of the condition and more encouraged that they could endure [48]. Thus, one of the factors that leads adolescents to romanticise mental health problems (the desire for social support), could also be seen as a beneficial effect of this practice—at least temporarily, for that individual at that moment in time. However, it is questionable whether this apparent benefit is truly useful for the young person in the long-term, given that romanticisation can also reinforce behavioural symptoms of mental health problems and discourage help-seeking from anyone other than the communities that romanticise the problem.

#### Directions for intervention

In the final component of this review, we examined whether any literature proposed intervention guidelines or strategies to reduce romanticisation and its associated harms. One proposed direction for reducing romanticisation was to prevent it spreading online by censoring romanticised posts, websites, forums, and hashtags. For example, a classification mechanism was designed on Tumblr to remove ‘deviant content’ (mental health-related posts that violate community guidelines—mostly pro-ED content or extremely graphic or triggering posts; [25, 28]. The mechanism compared pro-ED content removed by Tumblr moderators (the classification system already implemented on the site) with semantically similar posts, such as fitness and healthy dieting content. It then analysed new posts based on these comparisons, and combined this with manual input from ‘domain experts’ who understood Tumblr guidelines and pro-ED communities. Censoring these communities is still a topic of debate, as it is acknowledged as a necessary protective measure for vulnerable people but risks inflicting further stigmatisation on these communities [5], and removing their freedom of speech [27, 75]. In addition, such mechanisms can remove obviously graphic posts, but will likely be unable to remove more subtle forms of romanticisation (e.g. aesthetically-pleasing posts or messages offering admiration or approval).

In papers describing content analyses of social media or other online spaces, some general guidelines about reducing romanticisation are suggested. These guidelines analyses advise clinicians, schools and parents to gather more information on the internet activity of an adolescent, and to provide guidance to them on how to constructively use the internet, such as moderating social media usage (including knowing when to completely withdraw), paying less attention to likes and following, and having open discussion with trusted adults about their internet use [48, 89, 110]. Lastly, there were some suggestions that school-based interventions may be able to address the social basis for romanticisation, as can individual interventions that assess peer networks [2, 105]. Beyond this, however, there were few proposals in the literature for how to reduce romanticisation and its potential harms among adolescents, likely due to the lack of consistent research exploring what the phenomenon is and why it occurs.

## Discussion

This narrative review aimed to investigate romanticisation of mental health problems in adolescence, which we define as the unrealistic perception or presentation of mental health problems at this age as desirable or possessing any appealing qualities. The literature search identified 61 papers discussing instances of mental health problems being held in a positive regard, and covered a range of methodology, including quantitative and qualitative analyses of posts on social media, websites, forums, and news reports, self-report questionnaires, conceptual reviews and case studies. The review demonstrated that, when romanticised, adolescent mental health problems are portrayed as beautiful, glamorous or interesting, or as a useful means of achieving specific goals (particularly weight loss for eating disorders, and emotion regulation for self-harm behaviours). Romanticisation mostly occurred online, with frequent use of imagery, poetry, humour, and pop culture references. A number of reasons were proposed to explain why adolescents might romanticise mental health problems, including the increasing normalisation of mental health problems in society, and the potential social benefits and identity benefits that romanticisation might bring. Although a number of possible negative consequences of romanticisation were proposed, particularly a decrease in help seeking, there has been limited research into how romanticisation might be reduced.

There have recently been extensive attempts to reduce the stigma associated with mental health problems [102]. The current review indicates that it is crucial to understand whether, as stigma has been reduced, there may have been a concomitant rise in romanticisation. In other words, if the stigma of mental health problems is reduced, these problems may not only become neutral but in some cases may become desirable. Some researchers propose that when awareness is raised and stigma is reduced, mental health problems become normalised [13, 76]. Thus, perhaps de-stigmatisation attempts to make mental health problems seem more normal and human have turned them into a social norm instead (essentially, a behaviour we expect from one another). This would explain why more normalised mental illnesses like depression and anxiety are romanticised more than stigmatised disorders such as schizophrenia [44, 86, 87]. It is especially important to understand if adolescents are more susceptible to this romanticisation, since adolescents are more susceptible to peer influence [93], peer influence has been found to affect adolescent attitudes and stigma surrounding mental health [88], and adolescence is both a period of mental health risk and significant identity development [1, 16]. In addition to this, romanticised accounts of mental health problems appear in fictional entertainment aimed at adolescent audiences; it is therefore unsurprising if adolescents begin to identify with problems portrayed romantically via popular fictional characters.

It is important to note that, when mental health problems become normalised, they do not necessarily become romanticised or desirable. There is an assumption in the literature reviewed here that destigmatisation leads a mental health problem to be seen as desirable, but this is not necessarily true. Indeed, it may be helpful to view potential attitudes towards mental health problems as existing on a spectrum, from ‘stigmatised and undesirable’ at one extreme, ‘romanticised and desirable’ at the other extreme, but with a neutral attitude existing somewhere in the middle. This midpoint is likely the ideal viewpoint for individuals to hold, since it will be associated with benefits such as increased self-understanding, social support and help-seeking without the possible costs of romanticisation. It may be that it is possible for society to reach this ideal point of removing the stigma from a mental health problem, leading it to be viewed neutrally, without necessarily moving to the other extreme of that problem being romanticised. It is vital in future research to tease apart these distinct attitudinal phenomena, and to examine whether media literacy training can help teach young people to engage most critically and usefully with the mental health content they see, particularly online.

The current review highlighted the need for other further research. A key avenue for future research is to test the direction of the relationship between romanticisation and other variables, such as normalisation and destigmatisation, as discussed above, but also variables such as personal identification with mental health problems, and severity of mental health problems. This could be achieved with a combination of experimental paradigms and longitudinal designs. The research reviewed here were typically based on cross-sectional analyses of social media and website content that associate these factors with romanticisation, and often make the assumption that normalisation and romanticisation are equivalent concepts. In future research, experimental and longitudinal designs could assess the temporal relationship between destigmatisation, normalisation and identification with a mental health problem, and whether (and in what circumstances) this might lead to romanticisation, if at all. Future research could also assess associations between an individual adolescent’s romanticised attitudes towards mental health problems and that of their peers (using self-report measures such as the SOSS; [12]). If, as this review suggests, de-stigmatisation causes adolescents to view mental illnesses as a social norm that is desirable due to their wish to conform to their peers, we would expect romanticised mental health-related activity to increase in individuals if it increased among their peers. Equally, if mental health problems became normalised but not romanticised among peers, we might that individuals conform to these attitudes.

Future research should also examine whether romanticisation or its impacts, if they are indeed problematic, can be reduced. This is important, given that romanticisation might trivialise or misrepresent mental health problems [2, 35, 100] or discourage help-seeking [57, 112]. In the reviewed literature, recommendations to reduce romanticisation and its potential harms often involved increasing adults’ understanding of adolescent social networks and social influence, or implementing one-to-one or group interventions with adolescents themselves, particularly focusing on the role of romanticisation in identity formation [2, 52, 106]. Identification and disidentification with a mental health problem is a personal choice that clinicians cannot control; however, adolescents may benefit from additional support in understanding how their mental health problems relate to their sense of self, as this could reduce general distress, reduce romanticised beliefs that reinforce the problem, and prevent future distress should they choose to disidentify with their disorder.

### Review limitations

There are a number of limitations to this narrative review. The inconsistent terminology used in research investigating romanticisation, and the lack of reviews that bring this research together, means that some research may not have been identified with the search terms and therefore would not have been included for review. There are also limitations specific to content analyses of material on social media or the Internet, which was the methodology of several papers in this review. The search windows on Internet search engines, news databases or social media platforms can be extremely brief, sometimes lasting only 1 day [5, 22, 68]. Research findings can therefore become outdated and unrepresentative of current online environments very quickly, as posts, profiles, hashtags and even whole websites may be removed, deactivated or moderated after the data has been collected. Therefore, longitudinal content analyses may be more beneficial for observing and defining romanticisation in such fast-paced online environments [26, 28].

Online content included for analysis may also be specific to the country that the search was conducted in, as community guidelines and policies often change with location. Search engine and social media results often change based on the individual’s location and previous online activity [89], yet none of the articles discuss the potential effects this may have had on their research. Several papers returned from the literature search investigated romanticisation in non-Western adolescent samples; this small number of papers indicate that romanticisation of mental health problems may also occur in non-Western cultures, but were excluded for the purposes of this review—further research is required to assess the extent of any cross-cultural similarities or differences in romanticisation.

A final limitation of analysing social media and forum posts is that demographic information about individuals is often unavailable or incomplete, which may undermine the conclusions drawn about adolescents in this review. Posters’ age and gender were estimated in some cases where the information was not available [23, 36, 64]. If the age of most samples has been assumed or estimated, the conclusions drawn about adolescent romanticisation may not be accurate. Further research that combines self-reported demographic information and longitudinal content analysis of the person’s mental health-related online activity (for example, what material is posted, interacted with and searched for) could provide a more cohesive picture the individuals that romanticise online.

## Conclusions

This narrative review investigated the phenomenon of romanticisation of mental health problems in adolescence: how it is defined, why adolescents might do it, what implications this might have, and whether it can be reduced. Results demonstrated a range of phenomena that can be described as the perception or presentation of mental problems as desirable, indicating that romanticisation is a suitable term for this collection of phenomena and can be used as a standardised definition for future research. The review indicates that romanticisation of mental health problems is prevalent in social media, pop culture, news reports, and other internet spaces, particularly among adolescents. Romanticisation can trivialise mental health problems, isolate young people with these difficulties, and provoke judgement and mockery from others. On an individual level, adolescent identity formation appears to underlie many aspects of romanticisation; on a population level, efforts to de-stigmatisation and normalise mental health problems appear to contribute to romanticisation. Further research using combinations of self-reported measures and analyses of online mental-health related activity can provide clearer directions to intervene at these levels to reduce romanticisation and its associated harms.

## Data Availability

No datasets were generated or analysed during the current study.
